# Oligodendrocyte Physiology and Pathology Function

**DOI:** 10.3390/cells9092078

**Published:** 2020-09-11

**Authors:** Markus Kipp

**Affiliations:** 1 Institute of Anatomy, Rostock University Medical Center, Gertrudenstrasse 9, 18057 Rostock, Germany; markus.kipp@med.uni-rostock.de; Tel.: +49-381-494-8401; 2 Center for Transdisciplinary Neurosciences Rostock (CTNR), Rostock University Medical Center, Gelsheimer Strasse 20, 18147 Rostock, Germany

## Introduction

The adult vertebrate central nervous system (CNS) mainly consists of neurons, astrocytes, microglia cells and oligodendrocytes. Oligodendrocytes, the myelin-forming cells of the CNS, are subjected to cell stress and subsequent death in a number of metabolic or inflammatory disorders, among which is multiple sclerosis (MS) [1,2,3,4,5]. This disease is associated with the development of large demyelinated plaques, oligodendrocyte destruction and axonal degeneration [6,7], paralleled by the activation of astrocytes and microglia as well as the recruitment of peripheral immune cells to the site of tissue injury. Of note, viable oligodendrocytes and an intact myelin sheath are indispensable for neuronal health. For example, it has been shown that oligodendrocytes provide nutritional support to neurons [8], that fast axonal transport depends on proper oligodendrocyte function [9] and that mice deficient in mature myelin proteins eventually display severe neurodegeneration [10]. 

Due to the presence of multifocal white and grey matter demyelination in the CNS of MS patients, any pathogenetic concept has to provide an explanation for the highly specific destruction of myelin and oligodendrocytes. While several treatment options are currently available to dampen the peripheral, T- and B-cell driven inflammatory activity in MS patients, treatment options to ameliorate oligodendrocyte pathology and strengthen neuronal health are, unfortunately, limited. For the development of such novel therapies, a basic understanding of oligodendrocyte development, maintenance, destruction and regeneration is needed as well as novel tools to precisely monitor neuronal and functional deficits during pre-clinical studies. This Special Issue collects articles that address ongoing research into promoting myelin repair, address our understanding of the physiology and pathology of oligodendrocytes, summarize the interaction of oligodendrocytes with central and peripheral immune cells and introduce novel models that allow us to study oligodendrocyte physiology and pathology.

Therefore, various animal models exist with key characteristic features. In experimental autoimmune encephalomyelitis (EAE), the active or passive immunization with CNS-related antigens results in multifocal inflammatory CNS lesions with secondary oligodendrocyte injury and demyelination to a variable extent. Toxin models, such as the cuprizone model, are characterized by a primary oligodendrocyte degeneration leading to demyelination, axonal degeneration and reactive gliosis. The cuprizone model has become increasingly popular in recent years to study key pathological events during MS lesion development and progression. This Special Issue includes six articles using the cuprizone model to understand underlying MS pathologies.

To investigate mechanisms operant during de- and regeneration of the axon-oligodendrocyte-myelin compartment, and to develop effective MS treatment options, the following are required: (i) novel, dynamic technical platforms to investigate complex cell–cell interactions in a CNS-like microenvironment such as the oligodendrocyte-nanofiber platform described by Enz and colleagues [11]; (ii) unbiased evaluation systems to monitor disease progression and successful therapeutic interventions in pre-clinical models such as those described by Zhan an colleagues [12], Joost and colleagues [13], and Hochstrasser and colleagues [14]; and (iii) novel imaging modalities which would allow longitudinal studies as described by Khodanovich and colleagues [15]. Of note, a better understanding of the axon-oligodendrocyte-myelin compartment might lead to restorative therapies not just in MS but also other neuronal disorders such as Down Syndrome (reviewed by Reiche and colleagues [16]) or schizophrenia (reviewed by Raabe and colleagues [17]).

In this Special Issue, two papers address key regulators of oligodendrocyte development. Nocita and colleagues demonstrate, by using oligodendrocyte cell lines in combination with electrospun polystyrene microfibers as synthetic axons, that the pro-myelinating drugs Clobetasol and Gefitinib promote oligodendrocyte differentiation by G-protein-coupled seven-pass transmembrane receptor Smoothened (Smo) and EGFR/ErbB inhibition [18]. Another paper focuses on a rare genetic disorder called Williams–Beuren syndrome, which is caused by the deletion of genetic material from a specific region of chromosome 7. The deleted region includes up to 28 genes among the *Nsun5* gene, encoding a cytosine-5 RNA methyltransferase. This condition is characterized by mild to moderate intellectual disability or learning problems, unique personality characteristics, distinctive facial features and heart and blood vessel (cardiovascular) problems. The brains of patients suffering from Williams–Beuren syndrome show several oligodendrocyte-myelin abnormalities including a reduced myelin thickness, lower mature oligodendrocyte cell numbers and reduced mRNA levels of myelination-related genes. Yuan and colleagues report that a single-gene knockout of *Nsun5* in mice results in a reduced volume of the corpus callosum, paralleled by a decline in the number of myelinated axons and ultrastructural abnormalities of the myelin sheath [19]. Beyond this, the authors found that *Nsun5* was highly expressed in oligodendrocyte progenitor cells and *Nsun5*-KO mice show reduced oligodendrocyte progenitor cell proliferation, suggesting that *Nsun5* regulates the cell cycle in developing oligodendrocytes. 

Another protein highly expressed during oligodendrocyte development is the low-density lipoprotein receptor-related protein 1 (LRP1), a transmembrane receptor, mediating endocytosis and activating intracellular signaling cascades. Schäfer and colleagues generated a novel inducible conditional knockout mouse model, which enabled an NG2-restricted LRP1 deficiency [20]. Although the underlying pathways are not yet characterized, LRP1 appears to be a regulator of oligodendrocyte progenitor cell survival. 

The mechanisms underlying the progressive neurodegeneration in MS are currently unknown, but failure of remyelination appears to play a major role. Remyelination is a very complex biological process and can be classified, at the cellular level, as four consecutive steps: (i) proliferation of oligodendrocyte progenitor cells; (ii) oligodendrocyte progenitor cell migration towards the demyelinated axons; (iii) oligodendrocyte progenitor cell differentiation; and, finally, (iv) interaction of the premature oligodendrocyte with the naked axon (i.e., axon wrapping) [21]. The existence of so-called “shadow plaques” in post-mortem brains of MS patients, representing remyelinated lesions, clearly demonstrates that complete repair of MS plaques is principally possible, although it is more common to observe only limited repair at the edge of lesions [22,23]. It is not clear why in some patients remyelination is widespread while in others it is sparse, but aging might well play an important role. Gingele and colleagues demonstrate in their work, using the cuprizone model, that myelin repair and the repopulation of oligodendrocytes is less effective in aged compared to young mice [24], implicating that the regenerative potential of the CNS decreases during aging. Beyond this, this work provides a protocol to induce reproducible demyelination in aged mice, allowing the development of remyelination-promoting therapies in a disease-relevant experimental setting. By using the same model, Nyamoya and colleagues demonstrate that laquinimod, a substance previously shown to protect mature oligodendrocytes against metabolic insults, supports myelin repair in a non-supportive environment [25]. There is a growing list of drugs for relapsing remitting MS, and most of these drugs act by reducing the adaptive immune system. Treatments which promote remyelination would offer the potential to delay, prevent or reverse disability, and numerous pre-clinical as well as clinical studies currently focus on this highly relevant topic. 

To understand the physiology and pathology of the oligodendrocyte-myelin unit needs, on the one hand, a better understanding of the oligodendrocyte-intrinsic regulative pathways. On the other hand, cell–cell communication pathways are equally important. While two review articles of this Special Issue focus on the intrinsic regulatory networks of oligodendrocytes [26,27], Erik Nutma’s work focuses on the astrocyte–oligodendrocyte crosstalk [28]. In this review article, the authors nicely point out that communication occurs via direct cell–cell contact as well as via secreted cytokines, chemokines, exosomes and signaling molecules. Understanding the pathways involved in this crosstalk will reveal important insights into the pathogenesis and treatment of CNS diseases. One candidate protein implicated in this cell–cell communication network might be the Transient receptor potential ankyrin 1 (TRPA1) receptor, as described by Krizta and colleagues in this Issue [29]. The conditional deletion of *Trpa1* in *Gfap*-expressing astrocytes delayed toxin-induced demyelination in the cuprizone model.

Currently, MS is considered a multifactorial disorder, with substantial evidence for a role of both genetic and environmental factors. Several lifestyle changes might help to ameliorate the MS disease course, of which are physical and mental exercise [30], which can induce remyelination, or quitting smoking to ameliorate oxidative and nitroxidative stress [31]. In this context, the work published by Serdar and colleagues is of interest. The authors were able to demonstrate that caffeine and taurine, ingredients of energy drinks, induce degeneration of the axon-oligodendrocyte-myelin unit [32]. Considering the continuously rising number of children and adolescents consuming energy drinks, and the fact that brain development is vulnerable in this phase of life, a closer look at particular lifestyle changes might tell us a lot about MS and other neuronal disorders. 

The disease which comes first to our minds when thinking about oligodendrocyte pathology is, quite often, multiple sclerosis. As outlined, the destruction of the axon-oligodendrocyte-myelin unit is the key pathological feature of MS. However, several other diseases can be linked to oligodendrocyte pathology. Primarily, these are the leukodystrophies, a group of usually inherited disorders characterized by degeneration of the white matter in the brain. Examples are metachromatic leukodystrophies, Canavan disease or X-linked adrenoleukodystrophy. Beyond this, the de- and regeneration of oligodendrocytes appears to be an important pathological aspect of many other neuronal disorders including spinal cord injury, where remyelination improves functional recovery [33], Alzheimer’s disease, where myelination-related processes are recurrently perturbed in multiple cell types, suggesting that myelination has a key role in Alzheimer’s disease pathophysiology [34], or stroke, where it is believed that oligodendrocyte progenitor cells can promote angiogenesis [35]. Beyond this, it has recently been suggested that cells of the oligodendrocyte lineage can transform, under specific conditions, into antigen-presenting cells, suggesting that oligodendrocytes can act as active immunomodulators [36]. 

In summary, this Special Issue adds to our understanding of a central CNS cell population: oligodendrocytes. 
